# Association of *APEX1* and *XRCC1* Gene Polymorphisms With HIV-1 Infection Susceptibility and AIDS Progression in a Northern Chinese MSM Population

**DOI:** 10.3389/fgene.2022.861355

**Published:** 2022-03-16

**Authors:** Bangquan Liu, Kaili Wang, Jiawei Wu, Yuanting Hu, Xun Yang, Lidan Xu, Wenjing Sun, Xueyuan Jia, Jie Wu, Songbin Fu, Yuandong Qiao, Xuelong Zhang

**Affiliations:** ^1^ Key Laboratory of Preservation of Human Genetic Resources and Disease Control in China, Harbin Medical University, Ministry of Education, Harbin, China; ^2^ Laboratory of Medical Genetics, Harbin Medical University, Harbin, China; ^3^ The Second Hospital of Heilongjiang Province, Harbin, China; ^4^ College of Basic Medicine, Harbin Medical University-Daqing, Daqing, China

**Keywords:** human immunodeficiency virus, acquired immune deficiency syndrome, base excision repair, single nucleotide polymorphism, male and male homosexual transmission

## Abstract

**Background:** Some studies have shown that the base excision repair (BER) pathway has an effect on HIV-1 replication. *APEX1* and *XRCC1* as key BER genes may affect DNA repair capacity. However, the roles of single nucleotide polymorphisms (SNPs) in *APEX1* and *XRCC1* and their impact on HIV-1 infection and AIDS progression remain unclear.

**Methods:** A custom-designed 48-Plex SNPscan Kit was used for detection of single nucleotide polymorphisms. 601 HIV-1-infected men who have sex with men (MSM) and 624 age-matched healthy individuals were recruited in northern China. Four SNPs (rs1130409, rs1760944, rs2307486 and rs3136817) in *APEX1* gene and three SNPs (rs1001581, rs25487 and rs25489) in *XRCC1* gene were genotyped. The generalized multifactor dimension reduction (GMDR) method was used to identify the SNP-SNP interactions.

**Results:** In this study, rs1130409 G allele, rs1001581 C allele and rs25487 C allele were associated with a higher risk of HIV-1 infection susceptibility (*p* = 0.020, *p* = 0.007 and *p* = 0.032, respectively). The frequencies of *APEX1* haplotype TT and *XRCC1* haplotype CT showed significant differences between cases and controls (*p* = 0.0372 and *p* = 0.0189, respectively). Interestingly, stratified analysis showed that the frequency of rs1001581 C allele was significantly higher in AIDS patients with the CD4^+^ T-lymphocyte count <200 cells/μl than those with >200 cells/μl (*p* = 0.022). Moreover, significant gene-gene interactions among rs1130409, rs1001581 and rs25487 were identified by GMDR (*p* = 0.0107). Specially, individuals with five to six risk alleles have a higher susceptibility to HIV-1 infection than those with zero to two risk alleles (*p* < 0.001).

**Conclusion:** *APEX1* and *XRCC1* gene polymorphisms were associated with the susceptibility to HIV-1 infection and AIDS progression in MSM populations in northern China.

## Introduction

The spread of HIV-1 infection around the world has become a major global public health problem. HIV-1 virus is the pathogen of AIDS, which can infect human immune cells, causing immune system dysfunction and making the human body susceptible to a variety of diseases (Bandera et al., 2019). Several reports have shown that different heterogeneities of host genetics and host immune response contribute to different chronic complications and AIDS progression (O’Brien and Nelson, 2004; Speelmon et al., 2006).

DNA damage triggers genome instability, thereby burdening cells with dangerously disadvantageous mutations (Whitaker et al., 2017). Activated powerful DNA damage response (DDR) pathways through the DNA repair system, cell cycle checkpoints and cell death pathways to minimize the harmful effects of DNA damage. Base excision repair (BER), one of the primary DNA repair pathways, repairs the endogenous DNA damages including deaminations, depurinations, alkylations and a plethora of oxidative damages, and can enhance DNA single-strand break repair (SSBR) (Nemec et al., 2010; Hegde et al., 2012). Based on the multiple functions of DNA glycosylase, there are two classic BER pathways: short patch BER and long patch BER. APEX1 and XRCC1 are two key enzymes in short patch BER.

*APEX1* is a 2.21 kb gene located on chromosome 14q11.2-q12. It encodes an apurinic endonuclease, which recognizes and cleaves phosphodiester bonds through a hydrolysis mechanism, and specifically activates DNA damage repair (Parsons et al., 2004). *APEX1* polymorphism has been reported to be associated with the efficacy of platinum-based adjuvant chemotherapy on cervical cancer (Chung et al., 2006) and non-small cell lung cancer (Pérez-Ramírez et al., 2019). In addition, it may increase the risk of other cancers, such as breast cancer (Mitra et al., 2008), colorectal cancer (Wang L. et al., 2016) and pancreatic cancer (Chen et al., 2019).

The *XRCC1* gene is located on chromosome 19q13.2-13.3 and is about 33 kb long. In DNA damage repair, the encoded protein functions as a scaffold protein for specific repair enzymes and plays a role in the subsequent enzymatic steps (Caldecott, 2003; Caldecott, 2008). Polymorphisms of *XRCC1* may be involved in the pathogenesis of many diseases, including head and neck cancer (Nanda et al., 2018), diabetes (Wang et al., 2019) and Alzheimer’s disease (Qian et al., 2010). It has long been speculated that insertion of HIV-1 cDNA leads to the creation of DSBs and leaves single-stranded gaps connecting the viral and host DNA (Skalka and Katz, 2005). Effective repair of these lesions is critical to host cell integrity and subsequent transcription of the viral genome, and BER may play this role (Mbonye and Karn, 2017). Functional SNPs in the BER gene can affect gene expression or function, affect DNA repair ability, and eventually lead to cell dysfunction and mutagenesis (Hung et al., 2005).

In addition, it has been reported that BER may influence the sequence preference of HIV-1 integration site (Bennett et al., 2014). However, the association between BER gene polymorphisms and risk of HIV-1 infection has not been fully evaluated. Our previous works have confirmed that polymorphisms in mismatch repair gene MSH6 and homologous recombination gene MRE11 may play an important role in the development of AIDS (Liu C. et al., 2019; Wang C. et al., 2016). In this study, we investigated whether seven potentially functional SNPs in the *XRCC1* and *APEX1* genes are associated with susceptibility to HIV-1 infection and the AIDS progression in men who have sex with men (MSM) populations in northern China.

## Methods

### Subjects

In this case-control study, 601 HIV-1 positive males were recruited from the Center for Disease Control and Prevention of Heilongjiang Province in northern China. All patients had acquired HIV-1 infection through male and male homosexual transmission. We recruited 624 healthy men diagnosed as HIV-1 seronegative by comprehensive medical examination at the Second Affiliated Hospital of Harbin Medical University, as a control group. Based on age, 1:1 frequency matching was performed at 10-year intervals. The detailed characteristics of samples are shown in Table 1. The study was approved by the local ethics review committee, and all subjects signed written consent prior to the study.

**TABLE 1:** Characteristics of HIV-infected individuals and healthy controls.

Characteristics	Cases	Controls	*P* Value
	(*n* = 601)	(*n* = 624)	
Age range, years	16–75	16–75	
Mean age ±SD, years	33.81 ± 11.30	34.62 ± 11.89	0.2246^a^
Clinical stages, n (%)			
I	236 (0.393)	—	—
II	175 (0.292)	—	—
III	128 (0.213)	—	—
IV	60 (0.100)	—	—
CD4^+^ T cell counts (cells/μl), n (%)			
<200	90 (0.150)	—	—
200–500	298 (0.495)	—	—
>500	213 (0.355)	—	—

a Student’s *t*-test.

### SNPs Selection and Genotyping

Potential functional polymorphisms in the *XRCC1* and *APEX1* were identified through the dbSNP database (http://www.ncbi.nlm.nih.gov/) and HaploReg v4.1 (http://pubs.broadinstitute.org/mammals/haploreg/haploreg.php). With minor allele frequency (MAF) > 0.1 in CHB (Han Chinese in Beijing), four SNPs (rs1130409 T > G, rs1760944 T > G, rs2307486A > G and rs3136817 T > C) in *APEX1* gene and three SNPs (rs1001581 C > T, rs25487 T > C, rs25489 C > T) in *XRCC1* gene were finally selected. The candidate SNPs are located in the untranslated regions (5′-UTR, intron) and exon of genes, and there is no significant linkage disequilibrium (*R* ^2^ < 0.8) among these SNPs.

Following the manufacturer’s instructions, genomic DNA was extracted from 200 μl whole blood of each sample using the QIAamp blood kit (Qiagen, Hilden, Germany). Genotyping was performed using a customized 48-plex SNPscan kit (Genesky Biotechnologies Inc., Shanghai, China), which used double ligation and multiplex fluorescent PCR. For quality control, 5% of random control samples were genotyped twice to verify that the accuracy and repeatability of genotyping was 100%.

### Statistical Analysis

The Hardy-Weinberg equilibrium (HWE) of the control group was confirmed for each polymorphism by χ^2^ goodness-of-fit test. Two-sided chi-square test (adjusted chi-square test, yates for 1 ≤ T < 5) was used to compare allele and genotype frequencies between HIV-1 infected individuals and healthy controls. In association tests, odds ratios (ORs) and 95% confidence intervals (95% CI) were estimated as the relative risk associated with SNPs. The linkage disequilibrium (LD) and haplotype analysis was constructed by Haploview v.4.2 (http://sourceforge.net/projects/haploview/). Comparison of the CD4^+^ T-lymphocyte count in different genetic models was performed using Wilcoxon rank–sum test. Generalized multifactor dimensionality reduction (GMDR) (http://www.ssg.uab.edu/gmdr/) was used to analyze the gene-gene interaction models, and the best model was selected based on the consistency and accuracy test results of cross-validation. Association between the number of risk alleles and AIDS was calculated using logistic regression. Bonferroni correction was implemented to correct for multiple testing. Statistical analyses were performed using SPSS v.22.0 (IBM-SPSS, Inc., Chicago, IL, United States) and R statistical software (v3.6.3). *P* value <0.05 was considered statistically significant.

## Results

### Characteristics of Participants

A total of 601 AIDS patients and 624 healthy controls living in Heilongjiang Province were recruited in this study. Table 1 shows the basic characteristics of participants based on the CD4^+^ T-lymphocyte count and clinical staging. The mean ages of the patients and control subjects were 33.81 ± 11.30 and 34.62 ± 11.89 years, respectively. There was no significant difference in the distribution of age and gender between the two groups. The genotype distributions of candidate SNPs in control samples were in agreement with HWE (*p* > 0.05) (Table 2).

**TABLE 2:** Associations between *APEX1* and *XRCC1* gene polymorphisms and susceptibility to HIV-1 infection.

Genetic models	SNP alleles and genotypes	N (frequency)		*P* Value	OR (95% CI)
		Cases (*n* = 601)	Controls (*n* = 624)		
	*APEX1*-rs1130409 (HWE = 0.098)				
	Risk allele, G (%)	548 (0.456)	509 (0.409)	**0.020**	**1.210 (1.031–1.420)**
Codominant model (GG vs TT)	GG	121 (0.201)	94 (0.151)	**0.012**	**1.539 (1.100–2.152)**
Codominant model (TG vs TT)	TG	306 (0.509)	321 (0.515)	0.315	1.140 (0.883–1.471)
	TT	174 (0.290)	208 (0.334)	—	—
Dominant model (GG + TG vs TT)	GG + TG	427 (0.710)	415 (0.666)	0.094	1.230 (0.965–1.567)
Recessive model (GG vs TT + TG)	TT + TG	480 (0.799)	529 (0.849)	**0.020**	**1.419 (1.056–1.907)**
	*APEX1*-rs1760944 (HWE = 0.975)				
	Risk allele, G (%)	565 (0.470)	555 (0.446)	0.258	1.096 (0.934–1.285)
Codominant model (GG vs TT)	GG	139 (0.231)	124 (0.199)	0.213	1.223 (0.891–1.680)
Codominant model (TG vs TT)	TG	287 (0.478)	307 (0.494)	0.880	1.020 (0.786–1.324)
	TT	175 (0.291)	191 (0.307)	—	—
Dominant model (GG + TG vs TT)	GG + TG	426 (0.709)	431 (0.693)	0.544	1.079 (0.844–1.378)
Recessive model (GG vs TT + TG)	TT + TG	462 (0.769)	498 (0.801)	0.174	1.208 (0.920–1.588)
	*APEX1*-rs2307486 (HWE = 0.887)				
	Risk allele, A (%)	1140 (0.950)	1172 (0.941)	0.308	1.200 (0.846–1.702)
Codominant model (AA vs GG)	AA	541 (0.902)	551 (0.884)	0.986^a^	1.964 (0.186–20.772)
Codominant model (AG vs GG)	AG	58 (0.097)	70 (0.112)	0.861^a^	1.657 (0.15–18.296)
	GG	1 (0.002)	2 (0.003)	—	—
Dominant model (AA + AG vs GG)	AA + AG	599 (0.998)	621 (0.997)	0.974^a^	1.929 (0.182–20.446)
Recessive model (AA vs GG + AG)	GG + AG	59 (0.098)	72 (0.116)	0.330	1.198 (0.833–1.724)
	*APEX1*-rs3136817 (HWE = 0.914)				
	Risk allele, C (%)	136 (0.113)	114 (0.092)	0.079	1.265 (0.973–1.644)
Codominant model (CC vs TT)	CC	9 (0.015)	5 (0.008)	0.225	1.952 (0.662–5.754)
Codominant model (TC vs TT)	TC	118 (0.197)	104 (0.167)	0.163	1.231 (0.919-1.647)
	TT	473 (0.788)	513 (0.825)	—	—
Dominant model (CC + TC vs TT)	CC + TC	127 (0.212)	109 (0.175)	0.107	1.264 (0.951-1.679)
Recessive model (CC vs TT + TC)	TT + TC	591 (0.985)	617 (0.992)	0.253	1.879 (0.637–5.542)
	*XRCC1*-rs1001581 (HWE = 0.895)				
	Risk allele, C (%)	756 (0.629)	715 (0.576)	**0.007** **^b^**	**1.249 (1.062–1.469)**
Codominant model (CC vs TT)	CC	228 (0.379)	205 (0.330)	**0.003**	**1.691 (1.193–2.397)**
Codominant model (CT vs TT)	CT	300 (0.499)	305 (0.491)	**0.018**	**1.496 (1.070–2.090)**
	TT	73 (0.121)	111 (0.179)	—	—
Dominant model (CC + CT vs TT)	CC + CT	528 (0.879)	510 (0.821)	**0.005**	**1.574 (1.146–2.163)**
Recessive model (CC vs TT + CT)	TT + CT	373 (0.621)	416 (0.670)	0.072	1.240 (0.981–1.568)
	*XRCC1*-rs25487 (HWE = 0.176)				
	Risk allele, C (%)	910 (0.757)	887 (0.719)	**0.032**	**1.219 (1.017–1.461)**
Codominant model (CC vs TT)	CC	341 (0.567)	312 (0.506)	0.143	1.434 (0.885–2.325)
Codominant model (TC vs TT)	TC	228 (0.379)	263 (0.426)	0.607	1.138 (0.695–1.862)
	TT	32 (0.053)	42 (0.068)	—	—
Dominant model (CC + TC vs TT)	CC + TC	569 (0.947)	575 (0.932)	0.279	1.299 (0.809–2.085)
Recessive model (CC vs TT + TC)	TT + TC	260 (0.433)	305 (0.494)	**0.031**	**1.282 (1.023–1.607)**
	*XRCC1*-rs25489 (HWE = 0.302)				
	Risk allele, T (%)	136 (0.113)	113 (0.091)	0.066	1.279 (0.984–1.663)
Codominant model (TT vs CC)	TT	7 (0.012)	3 (0.005)	0.284^a^	2.536 (0.683–9.416)
Codominant model (CT vs CC)	CT	122 (0.203)	107 (0.172)	0.144	1.239 (0.929–1.653)
	CC	472 (0.785)	513 (0.823)	—	—
Dominant model (TT + CT vs CC)	TT + CT	129 (0.215)	110 (0.177)	0.093	1.275 (0.960–1.692)
Recessive model (TT vs CC + CT)	CC + CT	594 (0.988)	620 (0.995)	0.313^a^	2.435 (0.654–9.066)

Bold type indicates statistical significance (*p* < 0.05). OR: odds ratio; CI: confidence interval; HWE: Hardy-Weinberg equilibrium; SNP: single nucleotide polymorphism.

a Adjusted chi-square test, yates.

b *P* value remained significant after Bonferroni correction for multiple testing, in which *p* < 0.0071 (0.05/7).

### Association Between *APEX1* and *XRCC1* Gene Polymorphisms and Susceptibility to HIV-1 Infection

As shown in Table 2, the allele frequencies of *APEX1* rs1130409 and *XRCC1* rs1001581 and rs25487 were significantly different between the case and control groups (*p* < 0.05). Specially, the rs1130409 G (OR = 1.210, 95% CI = 1.031-1.420, *p* = 0.020), rs1001581 C (OR = 1.249, 95% CI = 1.062-1.469, *p* = 0.007) and rs25487 C (OR = 1.219, 95% CI = 1.017–1.461, *p* = 0.032) were associated with a higher risk of susceptibility to HIV-1 infection. In addition, the distribution of rs1001581 allele still showed significant differences between cases and controls after Bonferroni correction for multiple testing, in which *p <* 0.0071 (0.05/7) was set as statistically significant.

For the rs1130409, rs1001581 and rs25487 polymorphisms, there were significant differences in genotype distribution between HIV-1 infected group and control group under multiple genetic models (Table 2). In the analysis of *APEX1* rs1130409, genotype GG was associated with increased susceptibility to HIV-1 infection (recessive model: GG vs TT + TG: OR = 1.419, 95% CI = 1.056–1.907, *p* = 0.020; codominant model: GG vs TT: OR = 1.539, 95% CI = 1.100–2.152, *p* = 0.012). Additionally, *XRCC1* rs1001581 genotypes (CC + CT) were associated with increased susceptibility to HIV-1 infection (dominant model: CC + CT vs TT: OR = 1.574, 95% CI = 1.146-2.163, *p* = 0.005; codominant model: CC vs TT: OR = 1.691, 95% CI = 1.193–2.397, *p* = 0.003; CT vs TT: OR = 1.496, 95% CI = 1.070–2.090, *p* = 0.018). Moreover, the rs25487 CC genotype was also associated with increased susceptibility to HIV-1 infection (recessive model: CC vs TT + TC: OR = 1.282, 95% CI = 1.023–1.607, *p* = 0.031). However, after Bonferroni correction for multiple tests with *p* < 0.0018 (0.05/28), all these associations were no longer statistically significant.

### Haplotype Analysis

**FIGURE 1:**
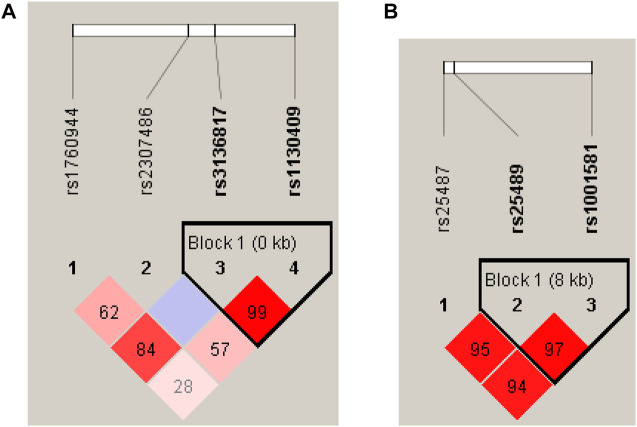
The results of LD analysis. **(A)** Haplotype block map for 4 SNPs of the *APEX1* gene, **(B)** Haplotype block map for 3 SNPs of the *XRCC1* gene. The haplotypes were constructed according to the prevalence of SNPs and LD among them. Numbers in squares indicate D’ values. The white cell indicates D’ < 1 and LOD (log of the likelihood odds ratio) < 2; The blue cell indicates D’ = 1 and LOD <2; the pink or red cell indicates D’ < 1 and LOD ≥2; The bright red cell indicates D’ = 1 and LOD ≥2.

Haploview software was used for linkage disequilibrium (LD) analysis. Two haplotype blocks were detected in *APEX1* and *XRCC1*. *APEX1* haplotype block1 is composed of rs3136817 and rs1130409 (Figure 1A), and *XRCC1* haplotype block1 is composed of rs25489 and rs1001581 (Figure 1B). After the *P* value calculated by multiple tests of 10,000 permutations, the frequencies of *APEX1* haplotype TT and *XRCC1* haplotype CT showed significant differences between cases and controls (*p* = 0.0372 and *p* = 0.0189, respectively) (Supplementary Table S1).

### Association Between *APEX1* and *XRCC1* Polymorphisms and AIDS Progressions

**FIGURE 2:**
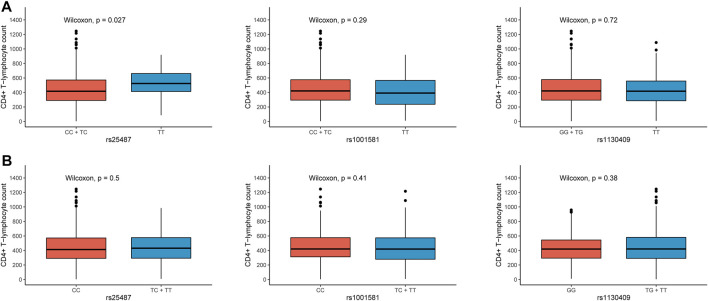
Differential analysis of CD4^+^ T-lymphocyte count in the different genetic models of rs25487, rs1001581 and rs1130409. **(A)** Dominant model, **(B)** Recessive model.

As the number of CD4^+^ T lymphocytes decreases, the immunity of HIV-1 infected patients will be severely impaired, leading to complications such as pneumonia and tumors. The CD4^+^ T-lymphocyte count was used as a surrogate marker for AIDS progression in our study. The frequency of rs1001581 C was significantly higher in the cases with the CD4^+^ T-lymphocyte count <200 cells/μl than those with >200 cells/μl (OR = 1.458, 95% CI = 1.056–2.012, *p* = 0.022) (Table 3). However, after Bonferroni correction for multiple tests with *p* < 0.0071 (0.05/7), all these associations were no longer statistically significant. We further found that patients with rs25487 CC and TC genotypes have a lower CD4^+^ T-lymphocyte count than those with TT genotype in the dominant model (*p* = 0.027) (Figure 2A), while there was no difference in CD4^+^ T-lymphocyte count in the recessive genetic model (Figure 2B).

**TABLE 3:** Association between the 7 SNPs and the clinical features of AIDS.

SNPs	Allele	CD4^+^ T-lymphocyte count, n (%)		*P*	OR (95% CI)	Clinical phase, n (%)		*P*	OR (95% CI)
		<200 cells/μl	>200 cells/μl			I + II + III	IV		
rs1130409	G	76 (0.432)	472 (0.461)	0.474	0.889 (0.644–1.227)	487 (0.450)	61 (0.508)	0.224	0.792 (0.543–1.154)
	T	100 (0.568)	552 (0.539)			595 (0.550)	59 (0.492)		
rs1760944	G	78 (0.443)	486 (0.475)	0.440	0.881 (0.639–1.215)	511 (0.472)	54 (0.450)	0.643	1.094 (0.749–1.598)
	T	98 (0.557)	538 (0.525)			571 (0.528)	66 (0.550)		
rs2307486	A	171 (0.972)	967 (0.946)	0.153	1.945 (0.780–4.849)	1023 (0.947)	117 (0.975)	0.185	0.460 (0.146–1.451)
	G	5 (0.028)	55 (0.054)			57 (0.053)	3 (0.025)		
rs3136817	C	25 (0.142)	111 (0.109)	0.197	1.359 (0.853–2.165)	120 (0.111)	16 (0.133)	0.466	0.813 (0.465–1.420)
	T	151 (0.858)	911 (0.891)			960 (0.889)	104 (0.867)		
rs1001581	C	79 (0.449)	367 (0.358)	**0.022**	**1.458 (1.056–2.012)**	686 (0.634)	70 (0.583)	0.276	1.237 (0.844–1.815)
	T	97 (0.551)	657 (0.642)			396 (0.366)	50 (0.417)		
rs25487	C	132 (0.75)	776 (0.758)	0.823	0.959 (0.664–1.385)	816 (0.754)	94 (0.783)	0.480	0.849 (0.538–1.338)
	T	44 (0.25)	248 (0.242)			266 (0.246)	26 (0.217)		
rs25489	C	157 (0.892)	907 (0.886)	0.807	1.066 (0.638–1.782)	963 (0.890)	103 (0.858)	0.299	1.336 (0.774–2.305)
	T	19 (0.108)	117 (0.114)			119 (0.110)	17 (0.142)		

Bold type indicates statistical significance (*p* < 0.05). OR: odds ratio; CI: confidence interval.

### Gene-gene Interactions Analysis

**FIGURE 3:**
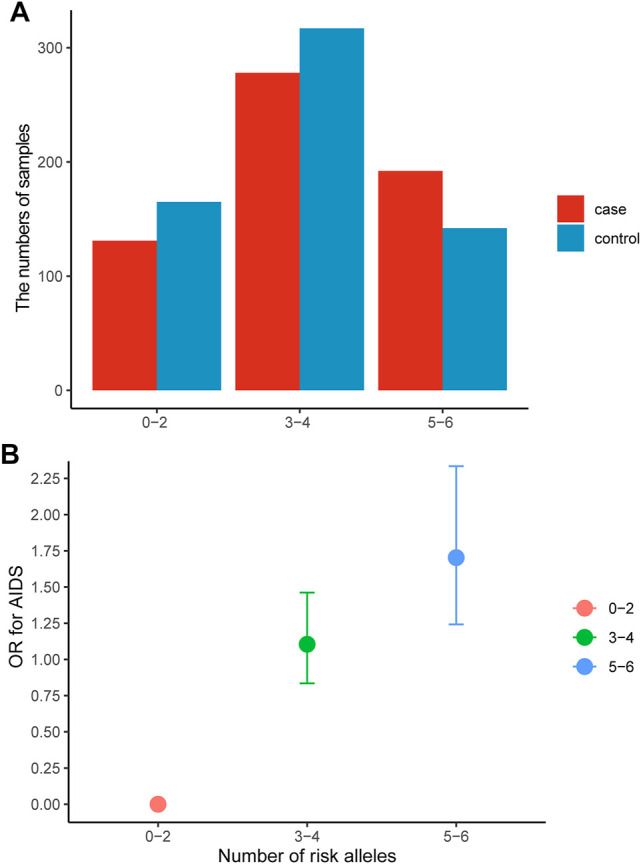
Association between the number of risk alleles in rs1130409, rs1001581 and rs25487 and AIDS. **(A)** the numbers of patients and healthy individuals with each risk allele in cases and controls, **(B)** The risk of AIDS by the number of risk allele categories. Data are crude ORs and 95% CIs. The 0–2 group was the reference. OR = odds ratio.

The GMDR model was employed to find the best SNP-SNP interaction combination among seven SNPs in *APEX1* and *XRCC1* genes. The results showed a significant two-locus model involving rs1130409 and rs1001581 (*p* = 0.0107) and a significant three-locus model involving rs1130409, rs1001581 and rs25487 (*p* = 0.0107) (Supplementary Table S2). The susceptibility to HIV-1 infection may be altered by the three genetic variants. We further assessed the association between the sum of risk alleles of rs1130409 G, rs1001581 C and rs25487 C and susceptibility to HIV-1 infection. Figure 3 A shows the numbers of patients and healthy individuals with each risk allele in cases and controls, and we observed an additive effect of risk alleles on the presence of AIDS (Figure 3B). The OR for AIDS was 1.10 (95% CI = 0.835–1.462) in patients with three to four risk alleles, and the corresponding OR in those with five to six risk alleles was 1.703 (95% CI = 1.242–2.335, *p* < 0.001).

## Discussion

It has been reported that the genetic background of the host will affect the susceptibility to HIV-1 infection and disease progression (Juno et al., 2012; Vidyant et al., 2017; de Silva et al., 2018). The interruption or dysregulation of SSBR can lead to genome instability, thereby promoting the integration of viral cDNA into the host genome (Sancar et al., 2004). One study also showed that the BER pathway is essential for effective lentiviral integration (Yoder et al., 2011). These findings suggest that the BER pathway may play a role in regulating HIV infection and integration. APEX1 and XRCC1 play a critical role in maintaining the integrity of the BER system. During the short patch BER process, APEX1 endonuclease cuts the 5′phosphodiester bond into an abasic site and recruits Polβ to fill the single nucleoside gap linked by the enzyme complex including LIG3 and XRCC1 (Izumi et al., 2000; Chatterjee and Walker, 2017). Therefore, this study aims to investigate the role of functional polymorphisms of *XRCC1* and *APEX1* genes in the susceptibility and clinical progression of HIV-1 infection, and to provide a theoretical basis for the prevention and treatment of the disease.

In our study, homozygous genotype GG and G allele of *APEX1* rs1130409 were found to be significantly associated with increased susceptibility to HIV-1 infection. The rs1130409 G allele has been reported to increase the risk of other diseases. Mitra et al. confirmed a statistical association between the rs1130409 G allele and breast cancer risk in north Indian women (Mitra et al., 2008). Recently, a study conducted by Usategui-Martín et al. showed a statistically significant association between Paget’s disease of bone and the rs1130409 G allele in Spanish (Usategui-Martín et al., 2018). rs1130409 in the *APEX1* exon 5 leads to the conversion of the residues at the carboxyl end of Asp148Glu, which may affect the endonuclease activity of APEX1. In addition, it has been reported that HIV-1 Rev protein interacts with APEX1 confirmed by *in vitro* binding experiments in HeLa cells (Naji et al., 2012). Yan et al. also demonstrated that siRNA-mediated knockdown of *APEX1* significantly reduced the level of HIV-1 cDNA integration and virus production in HeLa-CD4 cells, thereby inhibiting HIV-1 infection (Yan et al., 2009). The above functional studies on *APEX1* and rs1130409 can explain, to a certain extent, that individuals carrying rs1130409 G allele and GG genotype are more likely to be infected with HIV-1.

We also observed that homozygous genotype CC and C allele of *XRCC1* rs25487 were significantly associated with susceptibility to HIV-1 infection. Several studies found that the T allele or TT genotype were associated with the risk of a variety of cancers. For example, Liu et al. observed that rs25487 T allele significantly increased the risk of cervical cancer in the female population of southwest China (Liu G. C. et al., 2019). A study also showed that T allele of rs25487 was associated with poor prognosis of HBV-related hepatocellular carcinoma patients after hepatectomy (Yu et al., 2016). Mahmoud et al. suggested that rs25487 TT genotype could be regarded as possible genotypic risk factor for the development of HCV-related hepatocellular carcinoma (Mahmoud et al., 2019). Another study pointed out that patients with rs25487 genotype TT + CT had a higher risk of endometrial cancer in northern China (Chen et al., 2016). The results of the above association studies are inconsistent, which may be caused by the different disease types and populations used in the different studies. Moreover, we further found that patients with rs25487 CC and TC genotypes have a lower CD4^+^ T-lymphocyte count than those with TT genotype. rs25487 is a non-synonymous SNP, which changes the glutamine of *XRCC1* exon 10 into arginine, thereby reducing the affinity between XRCC1 protein and DNA repair complex and affecting the ability of DNA repair.

In addition, our results showed that homozygous genotype CC, heterozygous genotype CT and allele C of *XRCC1* rs1001581 were significantly associated with increased susceptibility to HIV-1 infection. The rs1001581 allele C still showed a significant association with susceptibility to HIV-1 infection, even after Bonferroni correction for multiple testing. Interestingly, there was a significant association between rs1001581 and CD4^+^ T-lymphocyte count, and the C allele could significantly accelerate the disease progression of AIDS. Gu et al. proved that rs1001581 C to T variation was significantly related to the protective effect of the Han population against HAV infection (Gu et al., 2018). Another study showed that rs1001581 heterozygous genotype CT was associated with the risk of advanced non-small cell lung cancer in Koreans (Kim et al., 2010). These results indicated that *XRCC1* rs1001581 may serve as an important biomarker for a variety of diseases and be beneficial for future evaluation of individual prognosis of AIDS. Although rs1001581 is located in *XRCC1* intron, it may affect the function of *XRCC1* encoding protein by altering its transcriptional activity. A functional study also reported that siRNA-mediated knockdown of *APEX1* and *XRCC1* can inhibit HIV infection in HeLa P4/ R5 cells (Espeseth et al., 2011).

AIDS is a consequence of the interaction between the individual’s genetic background and the environmental factors faced by the individual, in which gene-gene interaction may play a significant role. According to our GMDR model analysis, there was a significant SNP-SNP interaction between rs1130409, rs1001581 and rs25487. We observed a trend toward increased AIDS risk as the number of rs1130409 G, rs1001581 C and rs25487 C alleles increased, suggesting a cumulative effect of genetic variants on AIDS risk. Moreover, the association of haplotypes were analyzed to evaluate the synergy of SNPs. The frequencies of TT haplotype of *APEX1* and CT haplotype of *XRCC1* differed between cases and controls, and these associations were statistically significant. These results indicated that the interaction of rs1130409, rs1001581 and rs25487 significantly increased the risk of susceptibility to HIV-1 infection.

In the current study, we applied the Bonferroni correction to reduce the number of false positives in multiple significance testing. Although only the rs1001581 allele passed the calibration criteria after P-value was adjusted, the positive results of other SNPs should not be ignored. Undoubtedly, there are still some limitations in this study. First, there is a lack of information about critical factors in cases, including history of injection drug use, clinical data of viral loads and other clinical manifestations. Second, cases and controls were not exposed to the same conditions, and we did not collect blood samples from healthy MSM controls due to privacy regulations.

## Conclusion

In conclusion, the present study suggests that the polymorphisms of *APEX1* and *XRCC1* may increase the risk of HIV-1 susceptibility and the clinical progression of AIDS in MSM populations in northern China. In order to further understand the effect of BER gene polymorphism on AIDS, it is necessary to carry out functional studies and genetic association studies in different ethnic groups with larger samples.

## Data Availability

The original contributions presented in the study are included in the article/Supplementary Materials, further inquiries can be directed to the corresponding author.
