# Supplementary material for: Association of APEX1 and XRCC1 Gene Polymorphisms With HIV-1 Infection Susceptibility and AIDS Progression in a Northern Chinese MSM Population
Source: Front Genet. 2022 Mar 16;13:861355. doi: 10.3389/fgene.2022.861355 (PMC8966225; doi:10.3389/fgene.2022.861355)
Supplement: Supplementary file 1 [file Table1.DOCX]

**Supplementary Table S1.** The frequencies of haplotypes of the *APEX1* and *XRCC1* genes in case and control groups

| Haplotype | Frequencies | Haplotype frequencies  Cases Controls | | χ^2^ | *P* value |
| --- | --- | --- | --- | --- | --- |
| *APEX1*-block1 |  |  |  |  |  |
| TT | 0.568 | 0.544 | 0.592 | 5.664 | **0.0372^a^** |
| TG | 0.330 | 0.343 | 0.317 | 1.829 | 0.3464 |
| CG | 0.102 | 0.113 | 0.091 | 3.220 | 0.1623 |
| *XRCC1*-block1 |  |  |  |  |  |
| CC | 0.501 | 0.516 | 0.487 | 2.047 | 0.2994 |
| CT | 0.398 | 0.371 | 0.423 | 6.914 | **0.0189^a^** |
| TC | 0.101 | 0.113 | 0.090 | 3.584 | 0.1220 |

Bold type indicates statistical significance (*P* < 0.05).

^a^Haplotypes T_rs3136817_T_rs1130409_ and C_rs25489_T_rs1001581_ were supported after 10,000 permutations calculation *P* values in the Haploview program for multiple testing.
