# Supplementary material for: Association of APEX1 and XRCC1 Gene Polymorphisms With HIV-1 Infection Susceptibility and AIDS Progression in a Northern Chinese MSM Population
Source: Front Genet. 2022 Mar 16;13:861355. doi: 10.3389/fgene.2022.861355 (PMC8966225; doi:10.3389/fgene.2022.861355)
Supplement: Supplementary file 2 [file Table2.DOCX]

**Supplementary Table S2.** GMDR analysis for the best gene-gene interaction models

| Locus number | Best combination | Training  accuracy | Testing  accuracy | CV  Consistency | Sign Test(*P*) |
| --- | --- | --- | --- | --- | --- |
| 2 | rs1130409 rs1001581 | 0.5573 | 0.5309 | 10/10 | 9(**0.0107**) |
| 3 | rs1130409 rs1001581 rs25487 | 0.5714 | 0.5319 | 10/10 | 9(**0.0107**) |
| 4 | rs1130409 rs1760944 rs1001581 rs25487 | 0.5930 | 0.5024 | 6/10 | 6(0.3770) |

Bold type indicates statistical significance (*P* < 0.05). CV: cross validation; GMDR: generalized multifactor dimension reduction.
